# Talking placebo: a qualitative study of patients’ attitudes toward open-label placebo implementation into clinical practice

**DOI:** 10.3389/fpsyg.2025.1533663

**Published:** 2025-07-31

**Authors:** Antje Frey Nascimento, Berfin Bakis, Jens Gaab, Tobias Schneider, Athina Papadopoulou, Milena Ritter, Michael H. Bernstein, Charlotte R. Blease, Cosima Locher

**Affiliations:** ^1^Division of Clinical Psychology and Psychotherapy, Faculty of Psychology, University of Basel, Basel, Switzerland; ^2^Pain Unit, Clinic for Anesthesia, Intermediate Care, Prehospital Emergency Medicine and Pain Therapy, University Hospital Basel, Basel, Switzerland; ^3^Clinic of Neurology, University of Basel and University Hospital Basel, Basel, Switzerland; ^4^Department of Clinical Research, University of Basel, Basel, Switzerland; ^5^Department of Psychosomatics, University Hospital Basel, Basel, Switzerland; ^6^Department of Diagnostic Imaging, Warren Alpert School of Medicine of Brown University, Providence, RI, United States; ^7^Brown University Health, Rhode Island Hospital, Providence, RI, United States; ^8^Department of Women’s and Children’s Health, Uppsala University, Uppsala, Sweden; ^9^Digital Psychiatry, Department of Psychiatry, Beth Israel Deaconess Medical Center, Harvard Medical School, Boston, MA, United States; ^10^Department of Consultation-Liaison Psychiatry and Psychosomatic Medicine, University Hospital Zurich, University of Zurich, Zürich, Switzerland; ^11^Clinical Psychology and Psychosomatics, Faculty of Psychology, University of Basel, Basel, Switzerland

**Keywords:** open placebo, patients, attitudes, focus groups, qualitative research, patients and public involvement

## Abstract

**Background:**

For more than a decade, studies have supported the efficacy and safety of placebos without deception—so-called open-label placebos (OLPs)—to harness placebo effects in primary care while aligning with key ethical principles. Since treatment acceptance, feasibility, and successful implementation of novel interventions into clinical practice depend on patients’ attitudes, patients’ perspectives, perceived obstacles, and ideas on OLP use in clinical practice have yet to be elucidated. Therefore, patient and public involvement is increasingly demanded in research and its implementation into clinical practice. Qualitative research offers a unique opportunity to comprehensively understand attitudes, expectations, perceived benefits, and barriers from a patient’s point of view. Thus, we studied patients’ attitudes, concerns, and ideas toward OLP implementation into clinical practice with focus group discussions (FGDs).

**Methods:**

In 2022, three exploratory online FGDs, each including two patients with the same condition, were conducted with adult patients affected by chronic back pain (*n* = 2), chronic migraine (*n* = 2), or chemotherapy-induced emesis/nausea (*n* = 2). Physicians recruited participants in three outpatient clinics at the University Hospital Basel in Switzerland. The FGDs were held online for 60 min. Qualitative data was analyzed using *Reflexive Thematic Analysis,* applying an inductive-deductive hybrid approach within a social constructivist framework.

**Results:**

In total, five semantic-latent subthemes were identified, entailing: (i) *Placebos: Promising but risky*; (ii) *Acceptance of OLPs depends on a myriad*; (iii) *Be trustworthy, but deception may be necessary*; (iv) *Harnessing placebo effects without placebos*; (v) *From bench to bedside: Clinical transference of OLPs.* The themes reflect an in-depth discussion of the usage of OLPs in the clinical context, accompanied by different ambivalences regarding implementation, prerequisites, and the provider role.

**Conclusion:**

The FGDs provided insights into distinct attitudes, concerns, varying acceptance, and patients’ ideas regarding the clinical implementation of OLP interventions. While some patients displayed high acceptance, several concerns regarding ethical and practical issues have been expressed. OLP acceptance and attitudes toward practical issues of OLP intake differed between groups and within the same clinical condition.

**Trail registration:**

ClinicalTrials.gov, identifier NCT05166213.

## Introduction

A treatment approach that has received increased attention in the last decade is the open-label placebo (OLP) intervention. OLP interventions show a promising treatment alternative for a variety of disorders and complaints, comprising the transparent administration of placebo pills along with a treatment explanation (Buergler et al., 2023; von Wernsdorff et al., 2021). Deceptive placebos show robust effects for several psychological and physical health conditions (Kaptchuk and Miller, 2015), with clear neurobiological underpinnings, for example, for analgesic placebo effects by activating endogenous opioid, cannabinoid, and dopamine systems (Rossettini et al., 2023). Multiple randomized-controlled trials examined the efficacy of OLP interventions for conditions with substantial impairment, such as chronic back pain, migraine, irritable bowel syndrome, and cancer-related fatigue (Carvalho et al., 2016; Kam-Hansen et al., 2014; Lembo et al., 2021; Hoenemeyer et al., 2018). These studies reveal that OLPs not only reduce the level of suffering but also participants’ functional disability (Carvalho et al., 2016; Kaptchuk et al., 2010). The underlying mechanisms of change due to OLP interventions are still not fully understood, but factors such as patients’ belief system (Locher et al., 2021), faith in the treatment (Leibowitz et al., 2019), and empowerment (Locher et al., 2019) seem to be crucial components of the OLP effect. Taken together, this novel intervention approach without specific pharmaceutical ingredients, therefore without described side-effects, builds on harnessing general intervention context effects (Druart et al., 2023a) in a transparent manner by impacting expectations and conditioning processes (Wager and Atlas, 2015). The considerable effects of OLPs (Buergler et al., 2023), their interest in the research community (Kaptchuk, 2018), and the potential to harness placebo effects ethically introduce the question of how patients of the primary health system perceive the idea of implementing the OLP intervention in the real-world clinical setting (Blease et al., 2016).

Following this attempt, exploring the acceptability of OLP interventions for patients and health professionals is essential. Different studies examined doctors’ perspectives on placebo interventions (Bishop et al., 2014; Fent et al., 2011; Bernstein et al., 2020). A focus group study by Bernstein et al. (2020), encompassing two groups with 15 participants, indicated that physicians may have distinct opinions regarding applying OLPs in clinical practice: Some viewed OLPs as beneficial or harmless, while others considered OLP administration as a lack of respect toward patients. Still, general patients’ perspectives on using OLPs in clinical practice have rarely been examined (Locher et al., 2021; Bernstein et al., 2021; Haas et al., 2021), although doing so is crucial to enrich the debate about the ethical soundness and feasibility of OLP interventions (Blease et al., 2016; Blease et al., 2023; Blease et al., 2020) and to establish their feasibility in practice (Jones et al., 2023). Interestingly, a qualitative study from Switzerland showed that patients indicated more acceptance toward placebos than physicians would assume (Tandjung et al., 2014), whereas physicians and patients preferred placebos without deception (Ratnapalan et al., 2020). These findings also illustrate the awareness of the importance of actively involving patients is growing in clinical practice and research.

*Patients and public involvement* is a key requirement for sound practice in research and practitioners regarding implementing new approaches into clinical practice, yet rarely adopted (Hammoud et al., 2024). Research reveals that active patient engagement has the potential to bridge the gap between research and practice by making new interventions more feasible and acceptable (Domecq et al., 2014). This is particularly relevant for novel interventions, such as the OLP intervention, introduced into clinical settings, where understanding patient perceptions is essential (Bombard et al., 2018). In this context, qualitative research methods provide the opportunity to collect comprehensive information on constructs that fluctuate intrapersonally over time, such as attitudes and ideas (Braun and Clarke, 2006; Barbour, 2018). Qualitative research can ‘give voice’ to patients and tell their stories and perceptions in an organized way (Braun and Clarke, 2022). Further, they provide the opportunity to gather new information on affected patients’ ideas, highlight the practical implementation, and discuss perceived or assumed barriers (Barbour, 2018). Here, focus group discussions (FGD) are a promising way of assessing attitudes and ideas, in which the interaction of participants may create new ideas, perspectives, and concerns (Bernstein et al., 2020). FGDs efficiently elicit qualitative data from distinct participants and create data reflecting participants’ experiences, beliefs, and language (Wilkinson, 1998). Further, they allow researchers to observe interactions between participants and thus reflect patients’ understandings (Lehoux et al., 2006). What is more, FGDs have been shown to stimulate and encourage participants to develop debates about controversial topics (Barbour, 2018).

To explore what patients think about using OLP interventions in clinical practice and assess their ideas, degree of acceptance, and concerns, we conducted an exploratory focus group study with patients of the local University Hospital. An additional aim of this qualitative study was to examine the initial feasibility of recruitment supported by practitioners for a study on OLP implementation into clinical practice. To examine our study aims, we decided to include patients with distinctive conditions (i.e., chronic back pain, chronic migraine, chemotherapy-induced emesis, Parkinson’s disease, and women with menopausal complaints). The conditions were chosen based on the following criteria: The conditions had to reveal strong and clinically meaningful placebo responses (i.e., Shin et al., 2016; Quattrone et al., 2018: Parkinson’s Disease; van Lennep et al., 2021: chronic back pain) and/or they had to show promising OLP responses in previous trials (i.e., Kam-Hansen et al., 2014: migraine attacks; Hoenemeyer et al., 2018: cancer-related fatigue; Pan et al., 2020: women with menopausal complaints).

## Methods

### Study design

**Figure 1:**
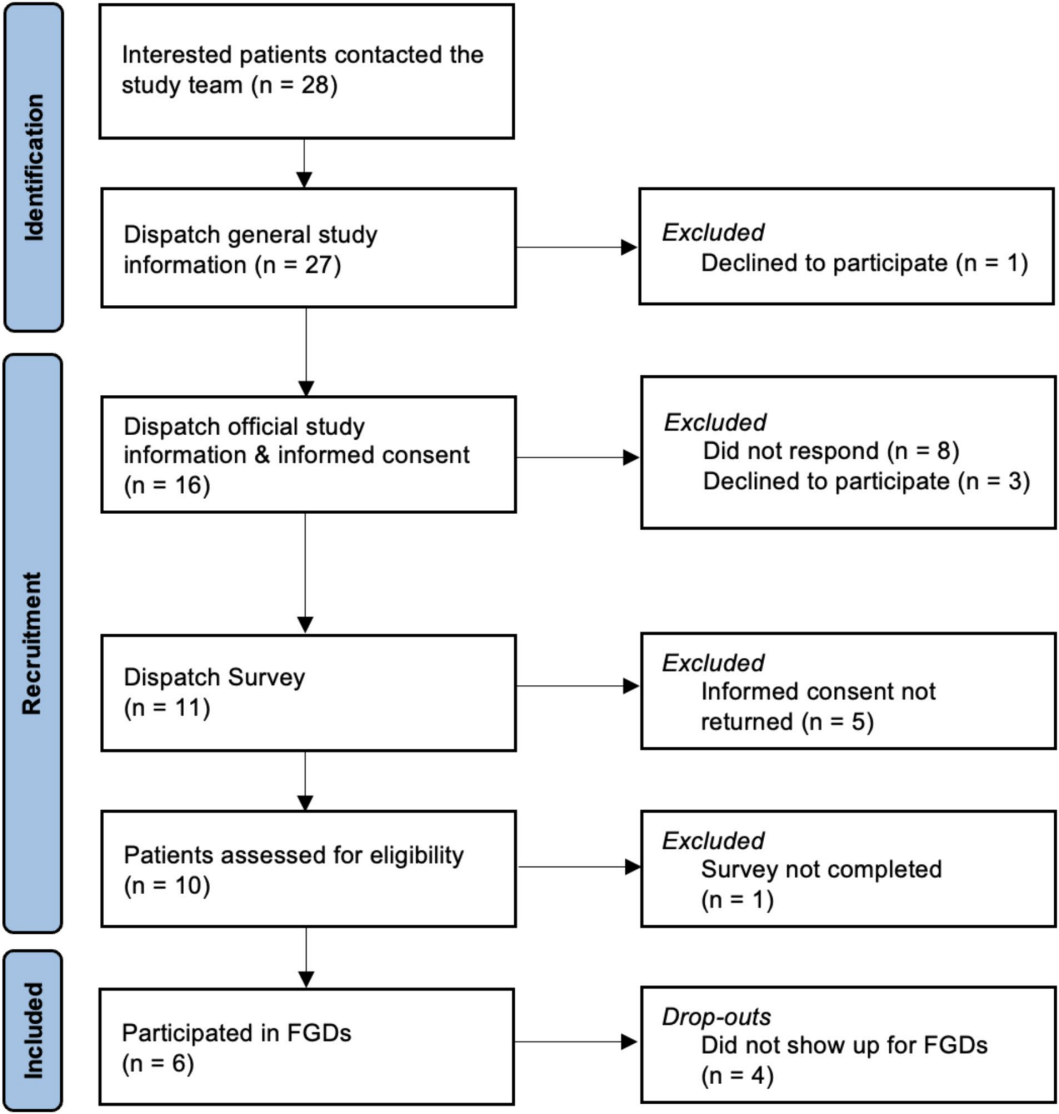
PRISMA study flow.

We conducted three exploratory online FGDs between September and November 2022 with different patient groups (in total *N* = 6) of the University Hospital Basel, Switzerland, affected by chronic back pain (*n* = 2), chronic migraine (*n* = 2), or chemotherapy-induced emesis/nausea (*n* = 2) to assess patients’ attitudes, concerns, and ideas on OLP implementation in clinical practice. Recruitment started in December 2021 and was aborted after no single patient with Parkinson’s Disease or menopausal complaints could be recruited in autumn 2023. Figure 1 presents the flow of participants. The recommended number of participants in focus groups remains a topic of discussion, with most experts suggesting groups of 5 to 10 participants (Carlsen and Glenton, 2011; Howard et al., 1989). Likewise, while there is no agreed-upon standard for the ideal number of focus groups in qualitative research, our objective was to conduct as many sessions as feasible.

Therefore, *a priori*, we had planned to conduct five FGD in total with around 5–8 participants in each group, including also patients affected by Parkinson’s disease and women with menopausal complaints, but we were not able to recruit patients from these two patient populations. Due to the COVID-19 pandemic and feasibility reasons, the FGDs were conducted online. We decided in advance to implement FGD when at least two participants would show up on the day of the FGD.

### Participants and recruitment

Participants were recruited through practitioners of the pain (TS), migraine (AP), and oncology (MR) outpatient clinic at the University Hospital Basel, Switzerland. Interested patients could contact the study team themselves or give permission to be contacted, after which they would receive a participant information sheet and an informed consent form. The study was advertised as “Open placebos in the clinical practice – your opinion is requested!” and described as having the aim to assess ‘patients’ opinions about placebos without deception in clinical practice.’ The flyer for our study can be found in Supplementary material. Participation in the whole study was compensated with 50 Swiss franc.

Different patient groups have been included to assess the attitudes and concerns of different clinical populations. The patient groups, respectively, the conditions were chosen based on already established susceptibility to placebo and OLP effects (Carvalho et al., 2016; Kam-Hansen et al., 2014; Hoenemeyer et al., 2018; Quinn and Colagiuri, 2015; Meissner et al., 2013; van Lennep et al., 2021). We aimed to include 5 participants (2 at minimum) per group for FGDs to allow a fluent online discussion, as limited group dynamics may occur in online FGDs with a larger number of participants.

Eligibility criteria included being at least 18 years of age, capable of consent and to attend an online conference due to the physical and psychological condition, and technical knowhow and equipment. Additionally, participants had to be affected by one of the following conditions: suffering from chronic back pain, chronic migraine, chemotherapy-induced emesis with the last occurrence at least before 14 days and at maximum before 1 year, Parkinson’s disease, or clinically significant menopausal complaints and being accompanied by a practitioner at the University Hospital Basel, Switzerland. After the informed consent obtainment, participants received a link to an online survey for screening purpose and assessment of sociodemographic data and characteristics of participants, including the transdiagnostic assessment of global burden of disease by self-report.

### Data collection and data analysis

Online FGDs were conducted via zoom.com with the primary investigator (AFN) and a facilitator of the study team (SI) to collect data on patients’ attitudes, concerns, and ideas regarding OLP implementation into clinical practice. Three FGDs were formed, including a group of patients with chronic back pain, chronic migraine, or chemotherapy-induced emesis/nausea. Each group consisted of 2 participants due to challenges in recruitment.

A semi-structured topic guideline with seven core questions was used to maintain consistency between focus group sessions and to assess answers regarding primary endpoints (see Table 1). A fictitious case vignette was presented at the beginning (see Supplementary material), followed by Question 1 to start the group discussion. Questions 1 and 2 assessed patients’ attitudes toward OLPs in clinical practice; Question 3 assessed the context in which patients would accept OLPs; Question 4 assessed patients’ concerns; Questions 5 to 7 assessed patients’ ideas for the clinical implication. The focus groups ended after 60 min, after the opportunity for the participants to anonymously document their thoughts and main messages via the website *padlet.com* (see Table 2).

**Table 1:** Questions of topic guide for focus group discussions.

Topic	Questions
1. Attitudes and context of acceptance	What do you think of the idea? What does this trigger in you? Under what circumstances would you personally take placebo pills, knowing that they are placebos?
2. Concerns	Why would you not take open placebos?
3. Disorder-specificity	Would it make a difference to you what kind of disorder someone has, e.g., depression instead of irritable bowel syndrome?
4. Trust	Would you trust a doctor who offered you a placebo treatment openly and without deception?
5. Ideas for clinical implication	What might an open placebo treatment look like there in the clinic? Where could placebos be given in the clinic, and how do you think a placebo treatment would work in the best-case scenario? What would be important from your point of view?

**Table 2:** Written final message of participants.

Migraine (*N* = 2)	Oncology (*N* = 2)	Pain (*N* = 2)
“Earlier I had a rejecting attitude toward open-label placebos. This did not change due to the online meeting.” “The discussion made me curious and open toward open-label placebos”	“Thank you for the possibility of participating in this discussion. I am ready to answer further questions.” “Discussion: I perceived the exchange as differentiated and open. The combination of knowledge and own opinion was valuable. From the discussion I mainly take with me the versatile opinions and thought-provoking impulses.” “Mutual attunement” “Topic of ‘positive psychology’” “Placebo in the sense of wholistic treatment, however only for the treatment of side-effects”	“Open Placebo Administration: Relationship between patient and doctor is indicative, setting, ‘ritual’, being taken seriously or not? Open, honest information [is] enormously important.” “One good thing about open placebo administration could be that the patient gains more confidence in the positive powers of their own body, if they can see it that way, that the actual work of reducing pain is done by them and not by a pharmaceutical. In this way, patients take on a more self-determined, active role again. I think that trust in one’s own body is sometimes disturbed in pain patients; one can even feel betrayed by the body. This could give them more self-confidence again.”

Final messages have been collected at the very end of the online focus group discussion anonymously using https://padlet.com. One message has been sent via email due to technical issues with the homepage.

The study plan has been submitted to the Ethical Committee of Northwestern and Central Switzerland (EKNZ ID Req-2021-01512), which decided with written confirmation that ethical review and approval were not required for this study. The study has been preregistered at clinicaltrials.gov (ClinicalTrials.gov NCT05166213).

### Qualitative data analysis

Recorded FGD were transcribed and anonymized. All identifying information was removed or changed ahead of analysis. The software MAXQDA2022 was used to analyze the data set using *reflexive thematic analysis*, according to Braun and Clarke (2022). Reflexive thematic analysis was chosen to retrieve an in-depth understanding of patients’ reality (Braun and Clarke, 2022). Data analysis was led by the first author (AFN) and supported by study team members (BB, CL), starting with independently reading the transcripts to achieve familiarization with the data. In the second step, a mainly inductive (i.e., data-driven) but also deductive (i.e., researcher-driven) open coding process was applied to identify code labels while looking for similarities, contradictions, and contestations across the data set within a social constructivist framework (Farvid et al., 2017).

We shared coding (AFN and BB) to achieve richer and more nuanced insights into our data set (Braun and Clarke, 2022). For passages that communicated numerous meanings, several codes were used. Then, codes with shared meaning were sorted into higher-level categories. This process yielded the final identification of themes, displaying the pattern of shared meanings across all three FGDs. The generation of themes and final analysis entailed mapping, revising, and discussing the results within the study team. Semantic (i.e., more descriptive) as well as latent themes (i.e., mirroring ideas, assumptions, or discourse) have been generated to enable a more thorough understanding of attitudes, concerns, and ideas of patients regarding open-label placebo implementation into clinical practice (Farvid et al., 2017). For the final in-depth data analyses, themes were conclusively defined and named, and related data excerpts representing the evolved themes were selected for presentation in the results section.

## Results

### Sociodemographic data

A total of six patients (five women; one man) participated in three FGDs. The mean (±SD) age was 47.4 (±9.47), ranging from 35 to 58 years. Most reported a higher education level and working part-time. Half of the participants were married and half divorced. Our sample encompassed predominantly moderate but also severely affected patients of three distinct clinical conditions: chronic pain, chronic migraine, or chemotherapy-induced emesis/nausea. All participants stated that they regularly take medication, and 40 medications have been mentioned, with the most frequent across all three groups being non-steroidal anti-inflammatory drugs. The second most mentioned medications were muscle relaxants, followed by antidepressants, immunosuppressants, and gastrointestinal medication. An overview of patient characteristics can be found in Table 3 and further details in Supplementary material.

**Table 3:** Sociodemographic characteristics of participants.

Sociodemographic characteristic	Overall (*N* = 6)		Chronic Back Pain (*n* = 2)		Chemotherapy induced Emesis (*n* = 2)		Migraine (*n* = 2)	
	*n*	%	*n*	%	*n*	%	*n*	%
Gender								
Female	5	83.3	2	100	1	50	2	100
Male	1	16.7	-	-	1	50	-	-
Civil status								
Single	-	-	-	-	-	-	-	-
Married	3	50	-	-	2	100	1	50
Divorced	3	50	2	50	-	-	1	50
Other	-	-	-	-	-	-	-	-
Highest educational level								
Middle school	1	16.7	1	50	-	-	-	-
High school	-	-	-	-	-	-	-	-
University or higher education	5	83.3	1	50	2	100	2	100
Employment								
Full-time	2	33.3	-	-	1	50	1	50
Part-time	4	66.7	2	100	1	50	1	50
Retired	-	-	-	-	-	-	-	-
PGI-S								
Normal	-	-	-	-	-	-	-	-
Minor	-	-	-	-	-	-	-	-
Moderate	5	83.3	2	100	1	50	2	100
Severe	1	16.7	-	-	1	50	-	-
Regular medication intake	6	100	2	100	2	100	2	100

PGI-S, Patient Global Impression of Severity of their condition.

All patients provided a placebo definition when asked in a survey ahead of the FGDs. All patients’ attitudes toward the term “placebo” were rated neutral, except for one, who perceived it as negative. None of the participants reported to have had a previous experience with placebos.

### Attitudes, concerns, and ideas regarding OLPs in clinical practice

Three exploratory online FGDs were concluded with two participants per group. Patients affected by chronic pain, chronic migraine, or chemotherapy-induced emesis/nausea expressed differentiated attitudes, concerns, and ideas regarding implementing OLPs into clinical practice. The majority mentioned concerns and an ambivalent attitude toward OLP. Ambivalences were expressed mainly regarding the cost–benefit balancing of OLP interventions and the issue of trust in providers. This is corroborated by the written messages of all patients expressed at the end of the online FGDs by anonymously posting summarizing thoughts or main messages via *padlet.com* (see Table 2). Notably, some participants had a clear position about OLPs: Whereas some participants stated that they would accept OLPs under certain circumstances; other participants fully rejected the idea of receiving OLPs.

**Figure 2:**
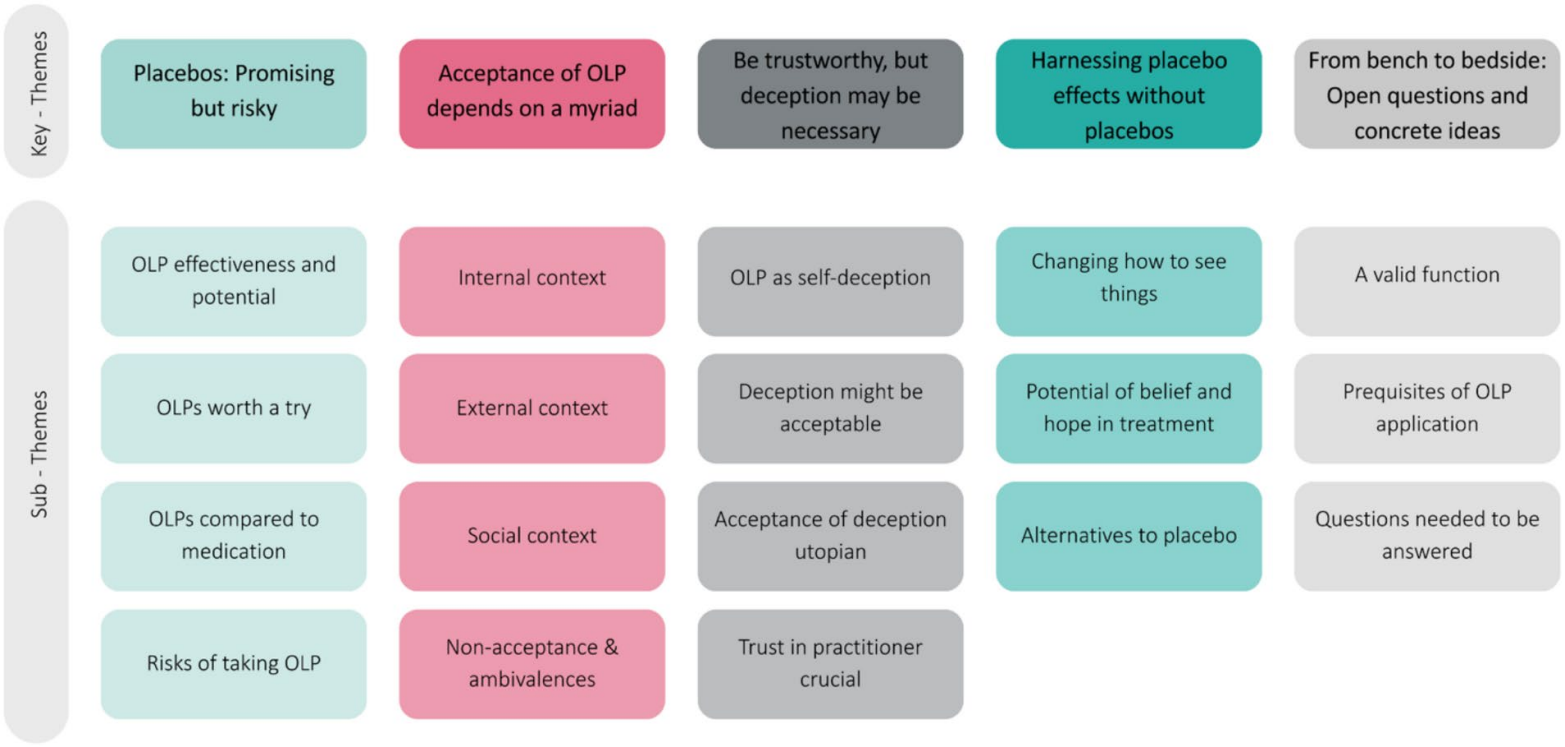
Overview of key- and sub-themes.

In total, five major themes were generated from the data set of our exploratory FGD encompassing different subthemes (see Figure 2 and Table 4 for an overview and description):

  i.  *Placebos: Promising but risky*

**Table 4:** Themes, subthemes, descriptions, and frequency.

Themes and subthemes	Subthemes	Description	Frequency (N of codes)
i) Placebos: Promising but risky!	*OLP effectiveness and potential* *OLPs: worth a try* *OLPs compared to medication* *Risks of taking OLPs*	Patients see benefits of OLP usage, but mention fear of disadvantages and potential dangers.	36
ii) Acceptance of OLPs depends on a myriad	*Internal context* *External context* *Social Context* *Non-acceptance & ambivalences*	A myriad of aspects has been mentioned to influence the decision to take placebos; as well as several open questions.	228
iii) Be trustworthy, but deception may be necessary	*OLP as self-deception* *Deception might be acceptable* *Acceptance of deception utopian* *Trust in practitioner crucial*	Principally the importance of transparency and trustworthiness of providers has been outlined. However, some patients mentioned to prefer deception along the placebo intervention.	66
iv) Harnessing placebo effects without placebo	*Changing how to see things* *Potential of belief and hope in treatment* *Alternatives to placebo*	Different possibilities have been mentioned by patients to elicit placebo effects without the necessity of a placebo agent.	25
v) *From bench to bedside: open questions and concrete ideas*	*A valid function* *Ideas of clinical application* *Prerequisites of OLP application* *Questions needed to be answered*	Patients reflected on possibilities how OLPs could be used in the clinical context but addressed different questions which should be answered ahead.	92

Data was used of the three conducted focus group discussions. Reflexive thematic analysis has been used for data analysis with semantic-latent and inductive-deductive coding, employing a social constructivist framework.

Patients displayed ambivalence by depicting that OLPs bear benefits but also mentioned the fear of disadvantages and harms. Subthemes included *OLP effectiveness and potential*, *OLPs worth a try*, *OLPs compared to medication,* and *Risks of taking OLPs*:

*OLP effectiveness and potential:* Participants shared their assumption that OLPs may work:

*“If someone were to give me something, I would probably feel an effect too <<laughs>>.” (FGD chronic migraine, A)*

*OLPs: worth a try:* Furthermore, the opinion was shared that it may be worth a try.

*“If my neurologist had said, ‘There is a new study, and it’s open placebo, but it still has an effect,’ then I would say, ‘Oh well, it does not hurt.’” (FGD chronic migraine, B),*

indicating that patients may try something new if recommended by practitioners, and it may be worth a try if it does not entail harm.

*OLPs compared to medication:* This openness to trying out a new approach comes along with worries and reported experiences concerning multiple risks medication can contain, such as the risk of addiction, limited effectiveness, or—several time mentioned—side effects:

*“I did not agree to it, I neither wanted to get an epilepsy medication, nor do I want to take any medications that can have side-effects like depression or stomach pain or whatever << laughs>>. Now I am back at the point where I…I also wrote to my neurologist, No, thank you. (…) I do not want to take any. I would rather take nothing than medication that has too many side-effects.” (FGD chronic migraine, B)*

*Risks of taking OLPs:* In contrast to a positive and open attitude, some participants also emphasized that OLPs and placebo interventions can be associated with danger. In more detail, concerns were expressed, such as being fooled by practitioners, fear of being disappointed, experiencing nocebo effects, and pressure of not being able to go back to treatment as usual if OLPs would not work. There was also the concern of being stigmatized and misjudged: If OLPs work, this could prove not having a “real” clinical condition. One participant also mentioned the fear of negative financial consequences from insurance companies, which might question patients’ strain fundamentally:

*“I am also curious about how insurance companies would react if it suddenly turned out that someone could live with a placebo. If it were beneficial for someone or if it could heal or improve psychological disorders. To what extent would an insurance company take you seriously? (FGD chronic back pain, C)*

Other adverse consequences, like dependency of OLPs, have also been mentioned:

*“Uhm, for me, it is important it doesn’t become something I fixate on. Because then it’s like a transfer of something; I believe that I need to be relatively independent in my pain. That’s not always possible. I also think that would be a bit of a hindrance for me, the drug, to engage with. Then it becomes almost again like a dependency – like I have to take it at five or something.” (FGD chronic pain, D)*

This main theme reflects patients’ ambivalence toward OLP benefits and unknown consequences of such a novel treatment approach.

  ii.  *Acceptance of OLPs depends on a myriad*

Several considerations have been mentioned to lead to an acceptance of an OLP intervention regarding the *internal, external, and social context*. However, also *non-acceptance* of OLPs and strong *ambivalences* have been expressed.

*Internal context:* To internal context belongs the patient’s persona with its attitudes, experiences, and condition, including the degree of strain and interference caused by the clinical condition.

*“… whether a depressed person would want to accept it or not. It probably just depends on how they deal with it and where they see the cause of their illness. And maybe one would need to clarify this VERY clearly with the patient themselves, why they BELIEVE that they have it.” (FGD chronic pain, D)*

*External context:* Influential aspects of the external context are mainly related to the practitioner and its openness toward OLPs:

*“To ensure a careful differentiation of benefits, effects, and expectations, this must be done very, very empathic and with great diligence [by the practitioner],” (FGD chemotherapy, E)*

Further aspects of the external context (i.e., the provider’s profession, time, place, treatment rationale, provided evidence, interdisciplinary interplay, and other treatment options) and the setting, such as in or outpatient setting, route of administration, the design of the placebo packaging, or the extent of the standard treatment regime, have been mentioned as well to influence the acceptance of OLP:

*“So I had a stem cell transplantation, and in the morning I had already to take 18 pills at once <<laughs>> and then the placebo on top of that would have been too much of a good thing.” (FGD chemotherapy, E)*

*Social Context:* A patient mentioned, for instance, receiving recommendations from a friend, but often, the relationship between practitioner and patient has been emphasized to be crucial for accepting OLPs:

*“(…) I can’t say directly yes or no. I think it also depends on how the doctor handles it, what kind of relationship I have with my doctor.” (FGD chronic pain, D)*

*Non-acceptance and ambivalences:* Single patients communicated clear rejection of the idea of OLPs. Especially in a ‘life or death situation’ when, for instance, patients have a cancer diagnosis, participants stated that OLPs would be no option. Patients also mentioned that even if OLPs would not have side effects and verum medication would have severe side effects, they would reject OLPs:

*“Well, for me, I must honestly admit, there are no circumstances to take a placebo.” (FGD chronic migraine, A);*

but different times patients communicated to feel ambivalent in regard of acceptance:

*“I am a bit ambivalent because I can’t really believe that it will actually work when I am a patient.” (FGD chemotherapy, F)*

This main theme includes most of the codes and shows a large array of aspects patients mentioned to affect their acceptance of OLPs in clinical practice. Also, this theme reflects strong ambivalence intra-and interpersonally in regard of OLPs.

  iii.  *Be trustworthy, but deception may be necessary*

Some patients mentioned that they would prefer to be deceived. At the same time, emphasized the need for a trustworthy practitioner and that OLPs should come from a practitioner they can trust. Interestingly, opinions diverged on whether the primary practitioner should offer OLPs or a different practitioner from the interdisciplinary team.

*OLP as self-deception:* Patients also reflected on whether OLPs could help to deceive themselves and are a form of deception:

*“What I can also imagine now is that someone is just used to taking medication all the time and…now they should slowly start to wean off a bit, but it’s like … a bit of self-deception, you know,” (FGD chronic pain, C)*

*Deception might be acceptable:* There was a clear ambivalence on the issue if deception might be acceptable. But patients mentioned that placebos should be given with deception and that the deception along with placebos might be even preferable:

*“So I would say, placebo treatment yes, … but only under the circumstances that it is not open. That [it] is not communicated,” (FGD chronic migraine, A)*

*Acceptance of deception utopian:* Whereas deception has been considered necessary by some patients and preferred in the context of a placebo intervention, ambiguously, there was consent that there would not be a situation where patients would agree with being actually deceived.

*“One never likes to be lied to. And this is always true in life, you know. It doesn’t really matter what topic it is, but in that case…”*

*Trust in practitioner crucial:*

*“And I believe it also depends on the trust relationship. So if placebos come into play after just the first or second conversation with the doctor, I might be more critical than if a long-standing relationship and trust have been established with a doctor.” (FGD chemotherapy, E)*

*“I simply must not feel like or receiving a feeling of being manipulated.” (FGD chronic pain, D)*

Hence, this theme clearly shows two positions of the participants: one of considering deception as a necessary mechanism of action of placebo effects, and another of advocating for transparency and trustworthiness in clinical practice.

  iv.  *Harnessing placebo effects without placebos*

Patients have mentioned different possibilities to elicit placebo effects without needing a placebo agent, including different strategies:

*Changing how to see things:* Cognitive strategies in coping with strain, by changing how to see things and applying mindfulness, were expressed multiple times.

*“(…) because you can embrace more and differentiate a bit more.” (FGD chemotherapy, E)*

*Potential of belief and hope in treatment:* Patients articulated that belief in treatments and hope may improve conditions:

*“But now not only with medications or pharmaceuticals, but in general. And what…, what the body does and achieves, just because one believes in it.” (FGD chronic migraine, B)*

*Alternatives to placebo:* Patients mentioned self-care, creativity, supplements, and rituals to be possibilities to harness placebo effects without a placebo agent.

*“What does it personally bring me? Because the medication [placebo] can’t provide me with anything directly. But do I experience an effect or something that feels good when I take the medication? It’s about self-care, taking time to do something good for me.” (FGD chemotherapy, E)*

Patients showed several ideas about how mechanisms of placebo effects might be beneficial without placebo agents.

  v.  *From bench to bedside: Open questions and concrete ideas*

Patients emphasized that the function of placebos should be clear if thinking about OLP transference in clinical practice and that different prerequisites of OLP interventions have to be fulfilled; however, many open questions came up that still would have to be answered to understand what OLP transference in clinical practice means and how it would look like.

*A valid function:* The function and precise aim of an OLP intervention has been questioned by participants:

*“Wouldn’t using a placebo be like patching a carpet? If you say you’re sitting at a computer all day, then the placebo, in my opinion, should address something and not just replace something like going outside… it should have its own place, you know what I mean?” (FGD chronic pain, D)*

*Ideas of clinical application:* Patients articulated different ideas of how OLPs could be used in the clinic. Positive framing of treatment effects instead of emphasizing negative adverse events, using placebos in the transition phase, substituting medication such as antidepressants with placebos, or using placebos for side effects together with a usual treatment regime, such as chemotherapy, were mentioned:

*“I had a donor transplant (…). Yes, it is… it is, honestly a difficult topic ‘placebo’ in this context. As I said, those are, well, side issues where diseases don’t hit as hard (…), rather [to] reduce certain symptoms. Then I am very open, I would be very open to placebo, but as I said (…) for the core disease, I would have more trouble with that. And I have to say, every morning, I am glad when I have 14 pills swallowed.” (FGD Chemotherapy F)*

*Prerequisites of OLP application*: Such as a careful consideration:

*“Then you would really have to take that apart carefully, um and, …and…, and…. <<laughs>>, yeah. So I wouldn’t just say YES, (…)” (FGD chronic pain, C)*

*Questions needed to be answered:* Patients raised several questions that have to be answered before implementing OLP in the clinic. In detail, the setting, expected efficacy, feasibility of transference into clinics, time frame of intake, and route of administration, among other points need to be defined. Also, the handling of the standard medication regime has been addressed:

*“Well, I don’t know either, you would have to stop EVERYTHING; I mean, I take several medications, so <<laughs>> YOU just have to stop EVERYTHING. Otherwise, you won’t know what works and what doesn’t, right?” (FGD chronic pain, C)*

This theme includes many still unanswered questions and requirements perceived by patients to implement the OLP in clinical practice.

### Initial feasibility of recruitment

Initially, we aimed to conduct five focus groups with a total of 25 participants. However, recruitment turned out to be much more challenging than anticipated. We were only able to conduct three focus group discussions with the *a priori* postulated minimum number of participants (i.e., total *N* = 6), participants affected by chronic pain (*n* = 2), migraine (*n* = 2), and chemotherapy-induced emesis/nausea (*n* = 2). This was despite the prior commitment of clinical partners to support our recruitment activities. There might be several reasons for our recruitment challenges, including reduced acceptability toward the OLP approach on the patients’ and/or practitioners’ side. Maybe the chosen patient populations were also less interested in participating in placebo research, given the high burden due to the chronic diseases, in which case studies around condition-specific medication or other topics might seem more relevant. Moreover, there might have been hurdles that are related to the placebo approach, such as reduced acceptability toward OLPs or the fact that placebos are not a disease-specific intervention. Finally, we conducted our study right after the COVID-19 pandemic, so practitioners might have been too occupied with pandemic challenges.

## Discussion

We explored patients’ attitudes, concerns, and ideas toward OLP implementation in clinical practice. In total, we conducted three FGDs with predominantly moderate but also severely affected patients of three distinct clinical conditions, i.e., chronic pain, chronic migraine, or chemotherapy-induced emesis/nausea. Our analyses yielded five main themes with subthemes around patients’ attitudes, concerns, and ideas: (i) *Placebos: Promising but risky*; (ii) *Acceptance of OLPs depends on a myriad*; (iii) *Be trustworthy, but deception may be necessary*; (iv) *Harnessing placebo effects without placebos*; and (v) *From bench to bedside: Clinical transference of OLPs.*

The first main theme of (i) *Placebos: Promising but risky* mirrors patients’ ambivalence to consider the OLP intervention—an unconventional novel approach—as promising and effective but at the same time considered as an intervention with risks, also entailing side effects (i.e., risk of addiction, stigmatization, limited effectiveness, etc.). Patients showed an attitude of ‘give it a try’ that has before been mentioned in other qualitative OLP studies (Locher et al., 2021; Kaptchuk et al., 2009) and has even been incorporated into the treatment rationale in clinical OLP trials (e.g., Nascimento et al., 2020). The shared perception of trying OLPs since they cannot harm stands in contrast to the many possible adverse consequences of taking OLPs patients mentioned as well, including concrete examples like potential impact on insurance cost coverage. The positive effects of OLPs, nevertheless, were underlined by considering OLPs an alternative to medication and the descriptions of multiple adverse events patients reported to have experienced with medication.

The second theme (ii) *Acceptance of OLPs depends on a myriad* delineates a plethora of aspects that patients mentioned to affect OLP acceptance related to different contexts. While some participants could, in general, or due to the severity of their personal condition, not imagine accepting OLPs, the severity of strain and the type of disease were mentioned to affect acceptance largely. Most frequently and mainly emphasized, the persona of the practitioner and the relationship with her or him have been mentioned to be crucial for acceptance, standing in line with research on the power of therapeutic relationships (Norcross and Lambert, 2019; Norcross and Wampold, 2018). This ambivalence was also observed in a focus group study of patients’ views of OLPs conducted by Druart et al. (2023a) in France. Since in only three FGDs with six patients in total, several components of the treatment context have been addressed to have a possible influence on acceptance and effects, we assume that patients are very aware of influential context factors in the therapeutic encounter and should be consulted as experts when it comes to implementing novel approaches into the clinical practice (see also Brett et al., 2014).

The third theme, (iii) *Trustworthy, but deception may be necessary,* reflects the ambivalence between the assumption that placebo effects are only elicited via deception and the widely perceived need for a trustworthy and transparent practitioner. The need for trust in practitioners, even if OLPs are prescribed, was also identified by Druart et al. (2023b). In our study, this ambivalence mirrors empirical challenges to the assumption that deception is necessary for placebo effects (Locher et al., 2017; Lembo et al., 2021; Buergler et al., 2023). Interestingly, patients described OLPs as working through self-deception. Hence, deceptive placebos include per definition deception by the practitioner, whereas OLPs may include self-deception. The ambivalence between voting for trustworthiness and transparency but preferring deception with placebo interventions may arise because patients are aware of the consequences of deception in clinical practice, potentially leading to relationship rapture with the practitioner. The urge of patients for trustworthiness is also incorporated into clinical practice standards because transparency is indispensable for sound therapeutic relationships, adherence to key ethical principles (i.e., respecting patient autonomy), and sound informed consent proceedings (Wear, 1998; Parascandola et al., 2002).

The fourth theme, (iv) *Harnessing placebo effects without placebos,* shows that patients know different possibilities to harness placeboid effects without placebo agents, including the importance of deliberate perception of strain, of hope and belief in improvement, and applying specific rituals, such as self-care, taking supplements, meditation, or yoga. This makes sense, given the rejecting attitude toward OLP interventions uttered by some patients in our FGDs. The theme *Acceptance of OLPs depends on a myriad* already displayed comprehensive ideas patients possess about relevant aspects of the intervention context and therapeutic encounter. The many possibilities mentioned to harness placebo effects without placebo products indicate that patients can find strategies to improve independently of placebo agents or specific interventions. Consequently, an active patient role possessing the possibility of improving one’s condition—at least partly—and caring for oneself has been underlined by patients. This vision of active and empowered patients aligns with the recent practice of perceiving patient engagement as a driving force toward improved clinical care, indicated by the increased application of shared decision-making in clinical practice (Söderholm Werkö, 2008; Hickmann et al., 2022; Covvey et al., 2019).

The fifth theme, (v) *From bench to bedside: Open questions and concrete ideas,* includes more practical thoughts and concrete ideas, but also many unanswered questions and requirements perceived by patients to implement OLPs in clinical practice. This aligns with the prevalence of still unanswered questions in the placebo research community regarding underlying mechanisms of action of OLPs, a precise differentiation from deceptive placebo responses, or an evidence-based treatment rationale (e.g., Blease et al., 2020; Kaptchuk, 2018). The concrete ideas participants mentioned of how placebos can be used may serve as starting points for OLP feasibility studies and warrant the implementation of OLPs for the usage in the transition phase of medication changes, substitution, or tapering, using OLPs for side effects together with a usual treatment regime, and focusing on positive framing of treatment effects instead of emphasizing adverse events.

We faced challenges in the recruitment process in clinical practice and were only able to conduct three out of five planned FGDs. This is a crucial learning relevant to the discussion on OLP implementation in clinical practice. Hence, our recruitment difficulties might be a significant indicator of the limited acceptability of the OLP approach – on the patient and practitioner side. This thesis has been discussed in other studies, including another exploratory FGD study that reported mixed opinions of practitioners toward OLPs, with some firmly rejecting OLPs as disrespectful to patients (Bernstein et al., 2020). Likewise, some studies found patients to be ambivalent toward the OLP approach. However, a part of the patients in our study indicated positive reactions, acceptability, and openness toward OLPs and placeboid effects (i.e., treatment context effects), in line with other previous studies that revealed good acceptability of the OLP interventions (e.g., Krockow et al., 2023).

To sum up, patients’ attitudes and acceptance toward OLP implementation into clinical practice were limited and ambivalent. Expressed concerns related to the issue of trust in providers, potential adverse consequences of taking OLPs, and open questions still needed to be clarified. Nevertheless, participants mentioned both ideas on how placebo effects can be harnessed without a placebo agent and concrete ideas under which conditions OLPs can be applied in clinical practice. These ideas may serve as starting points for implementing OLPs since these strategies appear warranted for affected patients who may benefit from such approaches.

### Theoretical and practical implications

Only three out of the five planned focus groups were conducted because not all practitioners provided sufficient support for recruitment. This lack of engagement suggests that implementing OLPs may also be challenging for practitioners. The next step should encompass further research, to see what barriers practitioners perceive in regard to harnessing placebo effects with OLP interventions. Thus, it might be necessary to conduct qualitative studies including interviews and FGDs with different health professionals regarding their attitudes toward OLP implementation into clinical practice, including also nurses who very often administer medication and accompany patients closely, while using already contextual factors deliberately to harness placebo and prevent nocebo effects (Palese et al., 2019). Yet, patients’ acceptance is a considerable prerequisite for implementing a new approach into clinical practice (Sekhon et al., 2017). Therefore, it seems necessary to address the open questions our participants asked related to the theme *From bench to bedside: open questions and concrete ideas* ahead of OLP implementation into clinical practice. Further, concrete ideas of patients could be considered as the first steps to use OLPs in clinical routine, such as OLP administration for the treatment of side effects or to taper medication like opioids.

The participants in our study mentioned diverse ways of *harnessing placebo effects without placebos*. Ideas of patients employing placebo effects to alleviate ailments and improve coping with daily stress can be starting points to create interventions harnessing placebo effects and show that patients themselves bear sufficient experiences and creativity to harness contextual placeboid effects.

### Strengths and Limitations

Our qualitative study has several limitations. First, the sample size was small, and all participants had similar sociodemographic backgrounds. Further, of the six participants, only one was male. The low participation rate could indicate less interest in placebo research or maybe low acceptance of OLPs. Thus, our results only allow very preliminary evidence for the potential benefits and perceived barriers of an OLP intervention in clinical practice, and the generalizability of the presented results is highly limited.

Each group consisted of two participants due to difficulties in recruitment and participation on the day the FGDs were conducted, which is below the usually recommended sample size for in-person FGDs of 5–13 persons (Matthews and Ross, 2010). Yet, the circumstance of only a few participants attending FGDs facilitated in-depth explorations of the *a priori*-defined questions in all three groups. Consequently, all questions of the topic guide (Table 1) could be addressed in all FGDs, and a broad array of issues has been raised and discussed between participants, also providing the space to share different personal information impacting thoughts and ideas on OLP administration in the clinical context. In contrast to quantitative techniques, qualitative methods are more case-related and provide more contextual information (Güthlin et al., 2008). To diminish potential biases due to the small sample size, we adhered strictly to a structured moderation protocol (i.e., the semi-structured guide) to ensure equitable opportunities for contribution for participants to reduce dominance bias. All FGDs had been conducted by the first author, reducing heterogeneity in the conduct, while documenting observations during and after the FGDs. Any peculiarities were discussed right after the FGDs among facilitators, entailing reflections on group dynamics. During the data analysis, diversity of perspectives was searched in the codes to ensure comprehensive coverage of the topic, and investigator triangulation was applied to ensure data interpretation from different angles.

Also, it is questionable if the online setting influenced our results, impacting group dynamics negatively. Yet, a positive effect might also be possible since participants could concentrate better on the verbal debate while feeling in a safe space in their known surroundings.

The fact that we conducted FGDs with patients is also an advantage of our study. Involving patients so that actual patient needs and preferences are understood leads to improved study designs and more relevant research questions and outcomes (Staniszewska et al., 2017). Patient involvement also enhances the ethical principles of research, emphasizing transparency and alignment with patients’ values (Brett et al., 2014). In clinical practice, patient involvement is crucial for implementing research findings into daily routine care since actively involving patients in clinical decision-making and treatment planning enables intervention adherence and higher-quality care (Domecq et al., 2014).

## Conclusion

Patients’ attitudes toward OLP implementation into clinical practice were ambivalent, and several concerns have been mentioned regarding the OLP intervention. Yet, patients shared different ideas of how to use placebo effects without placebo agents and how OLPs may find application in clinical practice, possibly serving as starting points for further feasibility studies and transference of OLPs into clinical practice.

## Data Availability

The raw data supporting the conclusions of this article will be made available by the authors upon resonable request.
