# Supplementary material for: Talking placebo: a qualitative study of patients’ attitudes toward open-label placebo implementation into clinical practice
Source: Front Psychol. 2025 Jul 31;16:1533663. doi: 10.3389/fpsyg.2025.1533663 (PMC12352116; doi:10.3389/fpsyg.2025.1533663)
Supplement: Supplementary file 1 [file Supplementary_file_1.docx]

**Supplementary**

This appendix has been provided by the authors to offer readers additional information about their work.

Supplement to: Frey Nascimento, Bakis, Gaab et al., Talking placebo: a qualitative study of patients’ attitudes towards open-label placebo implementation into clinical practice.

**Supplementary**

**Title:** Talking placebo: a qualitative study of patients’ attitudes towards open-label placebo

implementation into clinical practice.

Authors: Antje Frey Nascimento^1*^, Berfin Bakis^1^, Jens Gaab^1^, Tobias Schneider^2^, Athina Papadopoulou^3,4^, Milena Ritter^5^, Michael H. Bernstein^6,7^, Charlotte R. Blease^8,9^, and Cosima Locher^10, 11^

**Institutions:** ^1^ Division of Clinical Psychology and Psychotherapy, Faculty of Psychology, University of Basel, Switzerland; ^2^ Pain Unit, Clinic for Anesthesia, Intermediate Care, Prehospital Emergency Medicine and Pain Therapy, University Hospital Basel, Switzerland; ^3^ Clinic of Neurology, University of Basel and University Hospital Basel, Basel, Switzerland; ^4^ Department of Clinical Research, University of Basel, Basel, Switzerland; ^5^ Department of Psychosomatics, University Hospital Basel, Switzerland; ^6^ Department of Diagnostic Imaging, Warren Alpert School of Medicine of Brown University, USA; ^7^ Brown University Health, Rhode Island Hospital, Providence, RI, USA;^8^  Department of Women’s and Children’s Health, Uppsala University, Uppsala, Sweden; ^9^ Digital Psychiatry, Department of Psychiatry, Beth Israel Deaconess Medical Center, Harvard Medical School, Boston, MA, USA ; ^10^ Department of Consultation-Liaison Psychiatry and Psychosomatic Medicine, University Hospital Zurich, University of Zurich, Switzerland; ^11^ Clinical Psychology and Psychosomatics, Faculty of Psychology, University of Basel, Switzerland

**Correspondence:** Dr. Antje Frey Nascimento, Division of Clinical Psychology and Psychotherapy, Faculty of Psychology, University of Basel, Missionsstrasse 62, 4055 Basel, Switzerland. antje.freynascimento@unibas.ch

**Supplementary Appendix**

This appendix has been provided by the authors to offer readers additional information about their work.

**TABLE OF CONTENTS**

Section 1. Additional Details for Methods

Section 2. Additional Details for Results

**Section 1. Additional Details for Methods**

**Recruitment**

**Fig. 1 |** Recruitment Flyer.

**
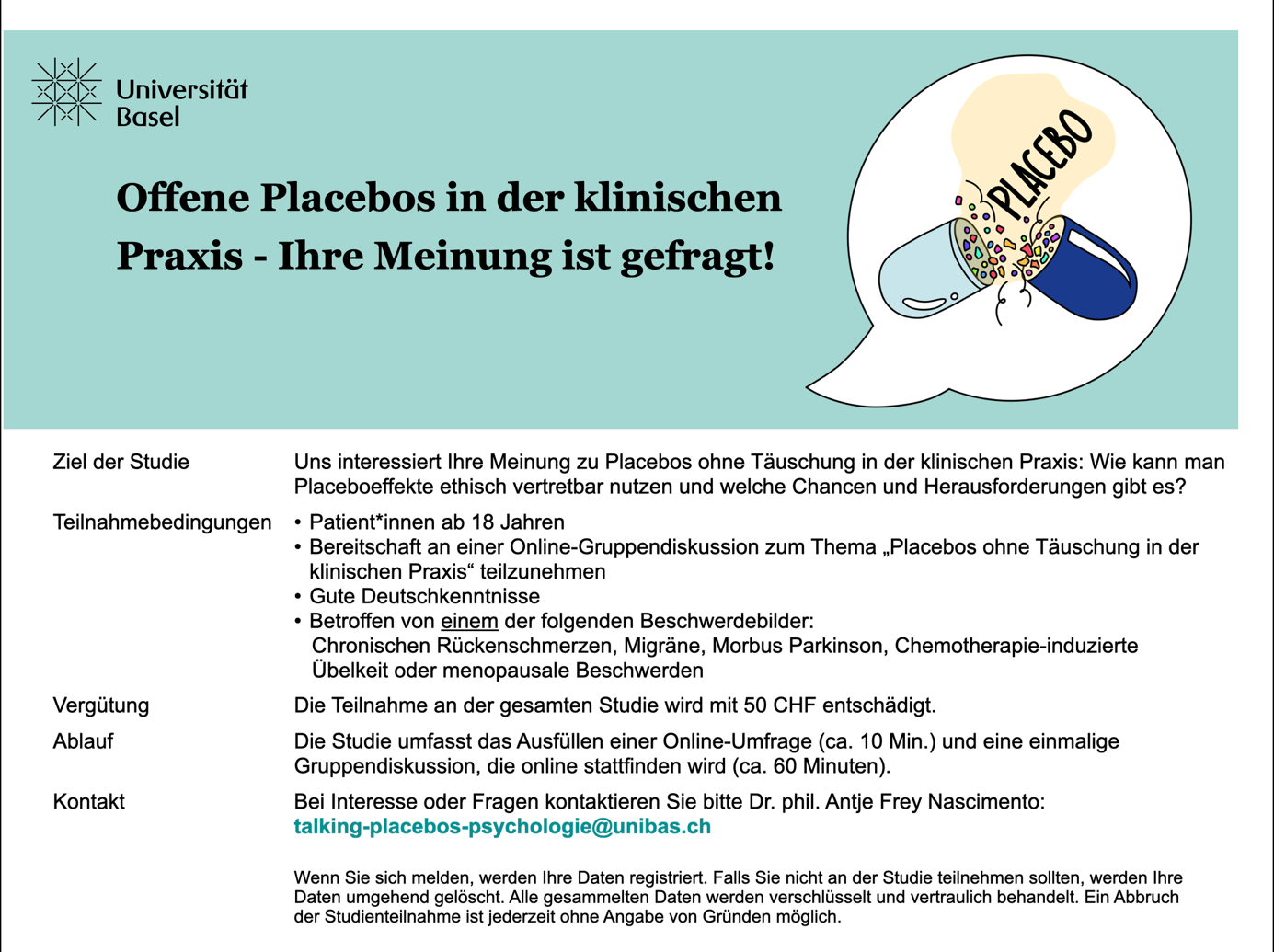
**

**Methods**

Script of Focus Group Discussions.

“Good morning/afternoon! I am happy to welcome you to our focus group discussion today. It’s great to have you here!

Today, I will ask you questions about placebos and their application in practice, which you can discuss here in the group. The focus will be on so-called "open" or "honest" placebos – meaning that patients are informed that they are receiving a placebo.

We are interested in your perspectives and thoughts. It is important to note that there are no right or wrong statements.

I am very grateful that you are participating in today’s session. As patients, I see you as experts in the treatment context. I am interested in what you think about placebos that are administered openly and without deception, whether you would accept them under certain circumstances, and what ideas you have on how to utilize placebo effects in practice.

To help me follow the discussion, I have SI with me today. If you need a short break during the discussion or if it becomes too much for you due to physical discomfort, you can message them directly or write in the group chat.

There are no specific rules for the discussion. It would be nice if everyone could say something on each point. Additionally, feel free to respond to the statements of other participants. It would help us if you pay attention to each other – this means allowing others to finish speaking and actively involving anyone who hasn’t yet contributed to a question.

As already mentioned, the discussion will be recorded. If you would like to make a comment later, please feel free to contact us again. During the discussion, I do not want to interrupt you, but it may be necessary for time reasons, and I would then address you directly.

Please speak in a way that you feel comfortable – Swiss German is also welcome. If you have questions regarding the open administration of placebos, I will answer them for methodological reasons only after the group discussion.

Do you have any questions? Otherwise, I will now start and am very curious about the discussion.

Introduction Rounds

Please set your view to gallery mode so that you can see all participants. I would ask you to briefly introduce yourselves – just state your name. (approx. 4 min.)

Brief Explanation

To ensure everyone has a comparable understanding of placebos, I would like to briefly explain what placebos are and what open and honest placebos entail:

Placebos look like real medications, are usually packaged as such, but contain no pharmacological active ingredients. In other words, there is no actual medicine in them, like a sugar pill. Here is an example [show P-dragees from Lichtenstein].

The effectiveness of placebos has been demonstrated for a variety of complaints. This means that people with complaints felt better after taking placebo tablets, and their symptoms actually decreased.

However, even when there is no deception, i.e., when it is openly stated that the intervention involves placebos, placebos can significantly alleviate symptoms. This has been shown, for example, in cases of chronic pain, irritable bowel syndrome, cancer-related fatigue, and menopausal symptoms.

Today, we will focus on this form of placebos: placebos that are administered openly and without deception.

Case Vignettes

I would now like to describe a sample situation of how an open and honest placebo treatment could look in practice:

Imagine a patient comes to their practitioner due to irritable bowel symptoms. The patient has been suffering from these symptoms for nearly 10 years, and the new medication they were supposed to take causes severe side effects. They do not feel well and want to discontinue treatment. However, the symptoms bother them a lot and interfere with many activities, such as work and socializing with friends.

The practitioner offers an alternative and says: “There exists also a completely different treatment approach. There are studies that show that irritable bowel syndrome complaints can be significantly reduced through placebo treatment, and this effect has also been found when study participants knew that they were receiving placebos. If you are interested, I am happy to tell you more about the open-label placebo intervention, especially why and how it can help. What do you think about that?”

**Section 2. Additional Details for Results**

Recent medication of included participants:

1. Analgesics

Non-Steroidal Anti-Inflammatory Drugs (NSAID)

Ibuprofen (2x)

Dexibuprofenum

Nopil (Naproxen)

Arcoxia (Etoricoxib)

Aspirin

Non-NSAID Pain Relievers:

Novalgin (Metamizole)

Paracetamol (if present in any over-the-counter combinations not listed here)

2. Opioids

Morphin (Morphine)

3. Muscle Relaxants

Mydocalm (Tolperisone) (N=2)

Sirdalud (Tizanidine)

4. Antidepressants and Mood Stabilizers

Trimipramin (Tricyclic Antidepressant)

Trittico 100 mg (Trazodone)

5. Anticonvulsants and Neuropathic Pain Agents

Pregabalini (Pregabalin)

Sanalepsi (Phenobarbital)

6. Antihistamines

Bilaxten (Bilastine)

7. Gastrointestinal Medications

Pantoprazol (Pantoprazole) (2x)

Ursofalk (Ursodeoxycholic Acid)

8. Immunosuppressants

Sandimmun (Cyclosporine)

CellCept 500 mg (Mycophenolate Mofetil)

Jakavi (Ruxolitinib)

9. Antivirals

Valtrex (Valacyclovir)

10. Antibiotics and Antifungals

Bactrim (Sulfamethoxazole and Trimethoprim)

Noxafil (Posaconazole)

11. Steroids and Corticosteroids

Prednison (Prednisone)

Hydrocortison (Hydrocortisone)

12. Beta Blockers

Bilol 10 (Bisoprolol)

13. Thyroid Medications

Eltroxin 0.05 (Levothyroxine)

14. Blood Pressure and Heart Medications

Amlodipin Eco (Amlodipine)

Zestril (Lisinopril)

15. Migraine Medication

Triptan (Triptan Class)

16. Bone Health Supplements

Calcimagon (Calcium and Vitamin D)

Kalicops D3 (Calcium and Vitamin D3)

17. Supplements

Acidum folicum 5 mg (Folic Acid)

18. Hormone Therapy

Climifemine (Estradiol)

19. Neuromuscular Blockers and Special Injections

Botox (Botulinum Toxin)

20. Cholinesterase Inhibitors (for Myasthenia Gravis)

Mestinon (Pyridostigmine)
